# Absolute and estimated values of macular pigment optical density in young and aged Asian participants with or without age-related macular degeneration

**DOI:** 10.1186/s12886-017-0557-5

**Published:** 2017-08-29

**Authors:** Yoko Ozawa, Yuta Shigeno, Norihiro Nagai, Misa Suzuki, Toshihide Kurihara, Sakiko Minami, Eri Hirano, Hajime Shinoda, Saori Kobayashi, Kazuo Tsubota

**Affiliations:** 1 Laboratory of Retinal Cell Biology, Department of Ophthalmology, Keio University School of Medicine, 35 Shinanomachi, Shinjuku-ku, Tokyo, 160-8582 Japan; 2 Department of Ophthalmology, Keio University School of Medicine, 35 Shinanomachi, Shinjuku-ku, Tokyo, 160-8582 Japan; 3 Wakasa Seikatsu Co., Ltd., 134 Chudoujiminami-cho, Shimogyo-ku, Kyoto, 600-8813 Japan

**Keywords:** Macular pigment, Retina, Lutein, Zeaxanthin, Age-related macular degeneration, Heterochromatic flicker photometry

## Abstract

**Background:**

Lutein and zeaxanthin are suggested micronutrient supplements to prevent the progression of age-related macular degeneration (AMD), a leading cause of blindness worldwide. To monitor the levels of lutein/zeaxanthin in the macula, macular pigment optical density (MPOD) is measured. A commercially available device (MPSII®, Elektron Technology, Switzerland), using technology based on heterochromatic flicker photometry, can measure both absolute and estimated values of MPOD. However, whether the estimated value is applicable to Asian individuals and/or AMD patients remains to be determined.

**Methods:**

The absolute and estimated values of MPOD were measured using the MPSII® device in 77 participants with a best-corrected visual acuity (BCVA) > 0.099 (logMAR score).

**Results:**

The studied eyes included 17 young (20–29 years) healthy, 26 aged (>50 years) healthy, 18 aged and AMD-fellow, and 16 aged AMD eyes. The mean BCVA among the groups were not significantly different. Both absolute and estimated values were measurable in all eyes of young healthy group. However, absolute values were measurable in only 57.7%, 66.7%, and 43.8%, of the aged healthy, AMD-fellow, and AMD groups, respectively, and 56.7% of the eyes included in the 3 aged groups. In contrast, the estimated value was measurable in 84.6%, 88.9% and 93.8% of the groups, respectively, and 88.3% of eyes in the pooled aged group. The estimated value was correlated with absolute value in individuals from all groups by Spearman’s correlation coefficient analyses (young healthy: R^2^ = 0.885, *P* = 0.0001; aged healthy: R^2^ = 0.765, *P* = 0.001; AMD-fellow: R^2^ = 0.851, *P* = 0.0001; and AMD: R^2^ = 0.860, *P* = 0.013). Using the estimated value, significantly lower MPOD values were found in aged AMD-related eyes, which included both AMD-fellow and AMD eyes, compared with aged healthy eyes by Student’s t-test (*P* = 0.02).

**Conclusions:**

Absolute, in contrast to estimated, value was measurable in a limited number of aged participants; however, it was correlated with estimated value both in young and aged Asian populations with or without AMD. These results may inform future clinical studies investigating the measurement of MPOD in understanding the role of macular pigments in the pathogenesis of AMD.

## Background

Among worldwide health concerns in aging populations is age-related macular degeneration (AMD), a leading cause of blindness in individuals older than 50 years of age [1, 2]. A preventive approach involving micronutrient supplementation based on results from large clinical studies, including the Age-related Eye Disease Study (AREDS) [3] and AREDS2 [4], is currently attracting attention. Micronutrient supplementation, including lutein/zeaxanthin, has been shown to attenuate the progression of AMD. Lutein/zeaxanthin are macular pigments as well as mesozeaxanthin, physiologically, and are believed to protect the macula and retina primarily through their anti-oxidative properties [5]. Previous reports have shown that oral intake of lutein from foods, serum concentration of lutein, and levels of lutein/zeaxanthin in the macula, can be measured and reflected in macular pigment optical density (MPOD), and are positively correlated, at least in healthy volunteers [6, 7]. However, it has also been proposed that MPOD is affected by several other factors, such as single nucleotide polymorphisms [8] and serum levels of oxidative low-density lipoprotein [6]. In addition, increases in MPOD are greater in individuals with a lower MPOD at baseline, suggesting that there may be a maximum tolerated dose in MPOD [9, 10]. Therefore, there may be differences in individual delivery and/or storage systems, and/or in the efficacy of additional lutein/zeaxanthin intake as micronutrient supplementation to foods. Thus, measuring MPOD may be important in monitoring and evaluating the efficacy of lutein/zeaxanthin uptake.

The controversial issue is that measurement methods of MPOD have not been fully developed or standardized, which has prompted current interest in both AMD clinicians and lutein/zeaxanthin researchers. Methods using Raman resonance spectroscopy [11–15] and autofluorescence spectrometry [16, 17] have previously been reported; however, these devices are not approved as medical devices nor are they widely available. In contrast, a technology based on heterochromatic flicker photometry (HFP) [18] has been developed, and a medical device, macular pigment screener, MPSII® (Elektron Technology, Switzerland) is now commercially available to clinicians and researchers. In this method, the difference in the intensity of blue (absorbed by the macular pigment) and green (not absorbed) wavelength flicker light that can be recognized in the fovea (where macular pigments accumulate) and para-fovea (where pigments are more scarce) is measured to estimate the level of macular pigment that blocks the blue wavelength of light and increases the threshold to recognize blue light by photoreceptor cells. Subtraction of the para-foveal threshold from the foveal threshold approximates the density of macular pigment. In this method, the effects of possible blue light blockade by intermediate bodies, such as cataracts, are subtracted. This is one of the advantages of the current model (MPSII®) compared with the previous model (M-POD®) manufactured by the same company [19]. In the previous model, only the foveal data were obtained; thus, the influence of cataracts was uncertain. Therefore, it would be important to assess the utility of the MPSII® device and, perhaps, adjust it for practical use.

Because obtaining both foveal and para-foveal data to calculate the absolute value of MPOD is a time-consuming process, the manufacturer proposes obtaining a more simplified value, namely, the estimated value of MPOD from only the foveal data with consideration of the subject’s age. The manufacturer developed a system to estimate the cataract concentration that affects the levels of blue light reaching the surface of the retina. Referring to data obtained from more than 5000 Caucasian individuals, the foveal data were then adjusted to develop an estimated value that is correlated to the absolute data in the population of their studies [20, 21]. However, whether these estimated values are applicable to Asian and, moreover, diseased eyes, remains unclear.

In the present study, we evaluated the use of the MPSII® device in an Asian population with or without AMD, to help optimize and establish MPOD values in a real-world, daily practice. We measured absolute and estimated values, and compared data from young and aged individuals with or without AMD, to determine the feasibility of using the MPSII® device in future clinical studies.

## Methods

The study adhered to the tenets of the Declaration of Helsinki, was approved by the Ethics Committee of the Keio University School of Medicine (Tokyo, Japan) (2010002), and was registered as UMIN000007649. Informed consent was obtained from all subjects.

### Subjects

This study was performed in the Department of Ophthalmology, Keio University School of Medicine from September 2013 to September 2016, and included 77 eyes of 77 participants whose best-corrected visual acuity (BCVA) was better than 0.8 in decimal score (0.099 in logMAR score). Seventeen eyes of 17 healthy participants who had no ocular disease except for refractive error, and 60 eyes of 60 outpatients referred to the hospital due to cataract and/or AMD and with BCVA better than 0.8 in decimal score (0.099 in logMAR score) were included. Individuals with high myopia (over −6.00D) were excluded. The patients were assessed using slit-lamp examination, and binocular indirect ophthalmoscopy after pupil dilation using 0.5% tropicamide. Patients who were suspected to have AMD in either eye according to binocular indirect ophthalmoscopy underwent examinations using fluorescein and indocyanine green angiographies and retinal camera (Topcon TRC 50DX, Topcon Corporation, Tokyo, Japan) and optical coherence tomography using a Heidelberg Spectralis OCT system (Heidelberg Engineering GmbH, Dossenheim, Germany) to diagnose AMD. All subjects underwent BCVA measurement using the refraction test.

### Measurement of MPOD

Both absolute and estimated values of MPOD were measured using a macular densitometer, macular pigment screener MPSII® (Elektron Technology, Switzerland), which uses an HFP technique in both eyes. The measurement was performed before pupil dilation and obtaining fundus images, and under non-mydriatic conditions. When measurement errors were obtained twice, the result was recorded as non-measurable.

### Statistical analysis

All results are expressed as mean ± standard deviation. Commercially available statistical software (SPSS version 23, Chicago, IL, USA) was used for analysis. Spearman’s correlation coefficient analyses and Student’s t-test were performed; differences were considered to be statistically significant at *P* < 0.05.

## Results

Seventy-seven eyes of 77 participants (38 male, 39 female; mean age 59.3 ± 20.0 years) whose BCVA was better than 0.8 in decimal score (0.099 in logMAR score) were analyzed. Among these, 17 (22%) eyes were included in the young healthy group; 26 (34%) eyes were included in the aged healthy (other than mild cataract) group; 18 eyes (23%) comprised the fellow eyes of unilateral AMD (AMD-fellow group); and 16 (20%) AMD eyes of patients who had bilateral AMD (AMD group) (Table 1). There were 2 pseudophakic eyes that were both included in the AMD group. The latter 3 groups included only those who were older 50 years of age, and had only mild cataracts or pseudophakia. Except for eyes in the AMD-fellow group, the eye with the better BCVA was studied. If BCVA was equal in both eyes, the eye with the better MPOD score, as measured using the MPSII® device, was assessed.

**Table 1:** Mean age and best-corrected visual acuity of the 4 groups

Groups	Eyes	Age	BCVA
Young healthy	17	25.5 ± 2.2	−0.08 ± 0.00
Aged heathy	26	71.0 ± 9.5	−0.02 ± 0.07
AMD-fellow	18	67.0 ± 9.1	−0.05 ± 0.06
AMD	16	67.5 ± 10.5	−0.04 ± 0.06

Data presented as mean ± standard deviation, unless otherwise indicated. BCVAs are shown in logMAR score. *BCVA* best-corrected visual acuity, *AMD* age-related macular degeneration

The mean age of each group was as follows: young healthy 25.5 ± 2.2 years; aged healthy 71.0 ± 9.5 years; AMD-fellow 67.0 ± 9.1 years; and AMD 67.5 ± 10.5 years. The respective mean BCVA (in logMAR score) were: −0.08 ± 0.00, −0.02 ± 0.07, −0.05 ± 0.06, and −0.04 ± 0.06. The young healthy group was significantly younger than the other 3 groups (*P* = 0.0001); however, there were no differences among the 3 aged groups, in which only those who were older than 50 years were included (data not shown). There were no statistically significant differences in mean BCVA among all groups (*P* = 0.15).

Subsequently, MPOD was measured using the MPSII® device. Both absolute and estimated values were measurable in all 17 (100.0%) eyes in the young healthy group; however, these values were measurable in only 15 of 26 (57.7%) eyes in the aged healthy group, 12 of 18 (66.7%) in the AMD-fellow group, and 7 of 12 (43.8%) in the AMD group (Table 2). Moreover, when the 3 aged groups were pooled, absolute value was measurable in 34 of 60 (56.7%) eyes in total (data not shown). In contrast, estimated value was measurable in 22 eyes (84.6%), 16 eyes (88.9%) and 15 eyes (93.8%), of the respective aged groups (Table 2). Estimated value was measurable in 53 of 60 (88.3%) eyes in the pooled aged group (data not shown).

**Table 2:** Number of measurable eyes and the mean values of macular pigment optical density

Groups	Absolute value		Estimated value	
	eyes (%)	mean ± SD	eyes (%)	mean ± SD
Young healthy	17 (100.0)	0.67 ± 0.13	17 (100.0)	0.75 ± 0.11
Aged heathy	15 (57.7)	0.61 ± 0.16	22 (84.6)	0.73 ± 0.16
AMD-fellow	12 (66.7)	0.49 ± 0.28	16 (88.9)	0.58 ± 0.24
AMD	7 (43.8)	0.65 ± 0.22	15 (93.8)	0.62 ± 0.19

*AMD* age-related macular degeneration, *SD* standard deviation

The mean absolute and estimated values that were measurable were 0.67 ± 0.13 and 0.75 ± 0.11 in the young healthy group; 0.61 ± 0.16 and 0.73 ± 0.16 in aged healthy group; 0.49 ± 0.28 and 0.58 ± 0.24 in the AMD-fellow group; and 0.65 ± 0.22 and 0.62 ± 0.19 in AMD group, respectively. There were no significant intergroup differences in either absolute or estimated values. Absolute values showed a trend toward lower values in the AMD-fellow group (*P* = 0.21), while the AMD group, in which 7 (43.8%) eyes were measurable, did not exhibit any trend (*P* = 1.00). Estimated values showed a trend toward lower values in the AMD-fellow group (*P* = 0.20) and AMD group (*P* = 0.58), although the trend in the AMD group was weak.

**Fig. 1:**
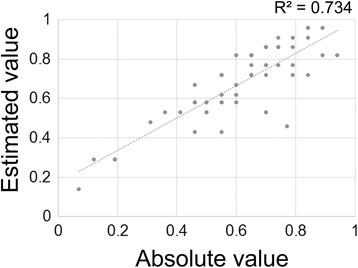
Correlation between absolute and estimated values of macular pigment optical density (MPOD). Absolute and estimated values of individuals in whom both absolute and estimated values were measurable are plotted. Spearman’s correlation coefficient analysis: *n* = 51; R^2^ = 0.734; *P* = 0.001

Then, to evaluate the feasibility of using estimated values as “official” data for MPOD measured using the HFP method, the relationship between absolute and estimated values in each group were analyzed (Table 3). In all 4 groups, there were significant correlations between absolute and estimated values in each individual (young healthy group: R^2^ = 0.885, *P* = 0.0001; aged healthy group: R^2^ = 0.765, *P* = 0.001; AMD-fellow group: R^2^ = 0.851, *P* = 0.0001; and AMD group: R^2^ = 0.860, *P* = 0.013). Moreover, the overall correlation between absolute or estimated values was also significant in all 51 participants in whom both values could be measured (Fig. 1) (R^2^ = 0.734, *P* = 0.001).

**Table 3:** Correlation between absolute and estimated values of macular pigment optical density

Groups	R^2^	*P* value
Young healthy	0.885	0.0001**
Aged heathy	0.765	0.001**
AMD-fellow	0.851	0.0001**
AMD	0.860	0.013*

Spearman’s correlation coefficient analysis. *AMD* age-related macular degeneration *P < 0.05, **P < 0.01

**Fig. 2:**
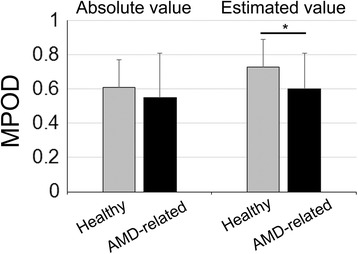
Estimated values in aged-healthy and aged-AMD-related eyes. Mean absolute and estimated values of aged and otherwise healthy group, and aged and with age-related macular degeneration (AMD) in the fellow or studied eye group were compared using the Student’s t-test. AMD-related group refers to pooled data from AMD-fellow and AMD groups. Absolute values (*n* = 15 and *n* = 19), and estimated values (*n* = 22 and *n* = 31); in aged healthy and aged AMD-related groups, respectively. Student’s t-test, absolute values: *P* = 0.44; estimated value: *P* = 0.02

Although differences in MPOD were not observed among the 4 groups, as mentioned above, the data from the aged healthy group and the pooled data from the other aged groups, including AMD-fellow and AMD eyes (aged and AMD-related eyes) were compared. No differences in the absolute values were measurable; however, estimated values showed significantly lower value in the latter group with AMD in the fellow or studied eyes (Fig. 2) (*P* = 0.02).

## Discussion

We demonstrated that MPOD could be measured using the HFP method, in which blue-green flickering light is stared at to determine the threshold of blue light recognition using the MPSII® device, in young and aged Asian participants with or without AMD. In young healthy patients, both values were measurable in 100% of the eyes. However, in the aged groups, absolute MPOD values were measurable in only 56.7%, while estimated values were measurable in 88.3%. The BCVA of all eyes was better than 0.8 (decimal score [0.099 in logMAR score]), and the average BCVA among the groups was not significantly different; nonetheless, there was difficulty in measuring the absolute value of MPOD in the aged groups. We assessed the estimated values and found that they were correlated with absolute values in individuals from all groups. Using the estimated value, significantly lower MPOD was demonstrated in aged and AMD-related eyes (i.e., pooled data from AMD-fellow and AMD groups), compared with the aged healthy group.

Although absolute values were measurable in all eyes of the young healthy participants, the proportion of eyes in which absolute values could be measured was less than two-thirds in all 3 aged groups with or without AMD. Moreover, when the 3 aged groups, aged otherwise healthy (other than mild cataract), aged and having AMD in the fellow eye, and aged having AMD in the studied eye groups were pooled, the absolute value measurement was successful in only 34 of 60 eyes (56.7%), and was still lower. However, estimated values were measurable in more than 80% of all the aged group, and in 56 (88.3%) eyes of the pooled aged group. This suggests that foveal value was measurable in many of the aged participants; the para-foveal value, however, was not (data not shown). To obtain para-foveal values requires staring at a dark point instead of a flickering light. Thus, it may be more difficult to obtain data from individuals with lower quality visual acuity compared with younger, healthier participants, regardless of whether their BCVA is better than 0.8 in decimal score (0.099 in logMAR score) and better than the allowance threshold required to have a driver’s license. Mild cataracts and AMD reduce the quality of vision, as previous reports have shown; these studies measured functional visual acuity [22, 23], which is a more sensitive measure of visual acuity [24, 25]. Alternatively, age itself may affect an individual’s endurance for concentrated focus on the dark stare point.

Under these conditions, if only absolute value, which adheres exactly to the concept of MPOD measurement, is analyzed in clinical studies, it may be possible to collect data from only a limited number of subjects because the procedure requires more time. Furthermore, data from aged eyes and/or with disorders may not be included because they are not measurable. Consequently, there would be a significant amount of missing data, and the relationship between MPOD, age and/or the disorders would remain unclarified or, at least, would be underestimated. In other words, the low data collection rate would severely impact study success.

In contrast, the estimated value, for which the influence of cataract was mitigated, was measurable in most of the participants, and also in aged eyes with or without AMD if, at least, they had satisfactory BCVA in the current study. Moreover, the AMD-related eyes exhibited lower levels of MPOD in estimated value, consistent with a previous report [12]. Eyes with AMD in the fellow eye may have a condition more closely approximating the AMD eyes than the otherwise healthy group (other than cataract), although the AMD lesion had not yet developed. In fact, AMD-fellow eyes are regarded as “risk eyes” [3, 4]. Measuring estimated values may enable the collection of more data from aged and/or AMD patients, which will enhance the study and contribute to a more profound understanding of pathogenic processes related to macular pigments.

However, the estimated value is determined only by foveal value and modified by age, according to the manufacture’s empirical algorithm based on the deduced age-related increase in the cataract measured using the Utrecht fundus reflection densitometer [20, 21] established before 1987 [20]. That study was conducted in the United States, and the data were likely obtained from a population of primarily healthy Caucasians.

Therefore, the important point is whether the estimated data are applicable to Asian populations as well as eyes with AMD and/or to an AMD-related eye, which has not been clarified. Although racial differences in cataract concentration between Caucasians and African Americans [26], and among Asian populations [27] have been assessed, differences between Caucasians and Asians have not been clarified. Moreover, eyes with AMD may exhibit a more aged condition, given that AMD is caused by cumulative oxidative stress, which causes accelerated aging of tissues [28, 29]. Before the current study, it was not clear whether the use of estimated values was feasible in human clinical studies involving an Asian population and AMD patients. To date, lutein levels and MPOD have attracted attention worldwide, and the results of the AREDS2 study [4] have been generally accepted, thus, further studies of MPOD are anticipated.

Limitations of the present study include the relatively small number of participants, and the involvement of 2 pseudophakic eyes in the AMD group, although 1 eye was non-measurable in both values, and the other eye was measurable only in estimated value; they did not have cataract, nevertheless, both showed similar results to others in the AMD group. The focus was exclusively on AMD; therefore, feasibility of the technique in other diseases remains unclear. Analyses of serum and dietary carotenoid levels were not performed. However, this was beyond the scope of the present study, but should be performed in the future and based on the current study, which demonstrated the feasibility of using estimated values in clinical studies.

In the present study, we evaluated absolute and estimated values acquired using the MPSII® device in young and aged Asians, with or without AMD. We found that absolute value could be obtained in a limited number of eyes in aged participants, in contrast to estimated values. Therefore, we also analyzed whether estimated values can replace absolute values which adhere to the original concept of HFP to measure MPOD, because significant amounts of missing data due to low data acquisition rates will hinder the use of the examination in clinical studies. Importantly, the estimated value was measurable in most of the participants and correlated well with the absolute value in all groups.

## Conclusions

Estimated values obtained using a commercially available device (MPSII®) were measurable in aged eyes with or without AMD-related lesions in an Asian population, and correlated with absolute values. These results may lead to increased use of the MPSII® device and, thus, contribute to the progress of future clinical studies investigating the impact of MPOD in the pathogenesis of AMD.

## Abbreviations

AMD: Age-related macular degeneration
AREDS: Age-related eye disease study
BCVA: Best-corrected visual acuity
HFP: Heterochromatic flicker photometry
MPOD: Macular pigment optical density
